# Giant schwannoma with extensive scalloping of the lumbar vertebral body treated with one-stage posterior surgery: a case report

**DOI:** 10.1186/1752-1947-8-421

**Published:** 2014-12-12

**Authors:** Yoichi Iizuka, Haku Iizuka, Ryoichi Kobayashi, Tokue Mieda, Kenji Takagishi

**Affiliations:** Department of Orthopaedic Surgery, Gunma University Graduate School of Medicine, 3-39-22, Showa, Maebashi, Gunma, 371-8511 Japan

**Keywords:** Giant cauda equina tumor, Posterior surgery, Scalloping lesion, Schwannoma, Transdural approach

## Abstract

**Introduction:**

Schwannoma is a relatively common benign spinal cord and/or cauda equina tumor; however, giant cauda equina schwannoma with extensive scalloping of the lumbar vertebral body is a rare pathology, and the treatment strategy, including the use of surgical procedures, is controversial. In this report, we present a rare case of a giant lumbar schwannoma of the cauda equina with extremely large scalloping of the vertebral body, and we discuss the surgical strategy we used to treat this pathology.

**Case presentation:**

A 42-year-old Japanese man presented to our department with complaints of a gait disturbance and muscle weakness in the left lower limb. His muscle strength in the proximal part of the left lower limb was grade 2 or 3/5, and he exhibited a mild urinary disturbance on the first visit. X-ray and computed tomography myelography of the lumbar spine showed an extremely large erosive lesion at the L3 vertebral body. Magnetic resonance imaging of the lumbar spine showed a large soft-tissue mass in the spinal canal at L2-L3 and the vertebral body at L3. A one-stage complete tumor resection and instrumented circumferential fusion were performed via a posterior approach, and a good outcome was achieved after the surgery.

**Conclusions:**

We performed one-stage posterior surgery in a patient with a giant cauda equina schwannoma with extensive scalloping of the vertebral body, and a good post-operative outcome was achieved.

## Introduction

Schwannoma is a relatively common benign tumor that can arise from the cranial or peripheral nerves, including the spinal nerves; however, giant cauda equina schwannoma is an uncommon pathology. In particular, giant lumbar schwannoma of the cauda equina is very rare compared to sacral lesions [1–10]. Moreover, spinal nerve schwannomas are sometimes observed as a pathology with erosive lesions in the vertebral body, so-called *scalloping*, and in rare cases the lesions become extremely large a long time after onset [11]. In this report, we present the case of a patient with giant lumbar schwannoma of the cauda equina, which we successfully treated with one-stage complete tumor resection and circumferential fusion via a posterior approach. We also discuss the use of surgery for treating this pathology.

## Case presentation

**Figure 1:**
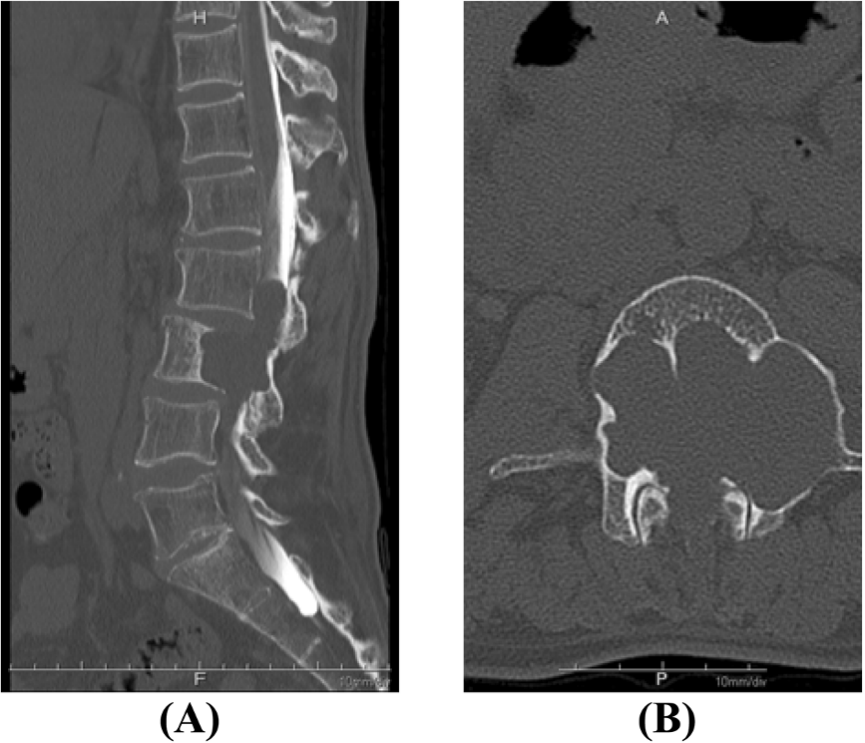
**Large scalloping of the L3 vertebral body.** Pre-operative sagittal **(A)** and axial **(B)** computed tomography myelography scans of the lumbar spine show extremely extensive scalloping of the L3 vertebral body.

**Figure 2:**
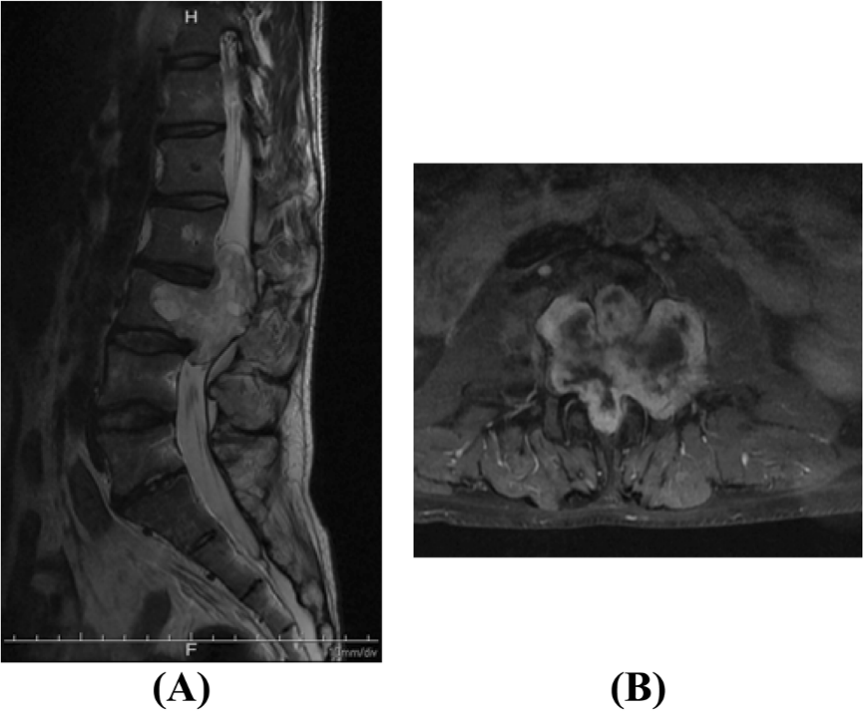
**Pre-operative magnetic resonance imaging scans of the lumbar spine.** A large tumor mass in the spinal canal at L2-L3 and the vertebral body at L3 can be visualized with increased signal intensity on a sagittal T2-weighted image **(A)** and a gadolinium-enhanced T1-weighted image **(B)**.

**Figure 3:**
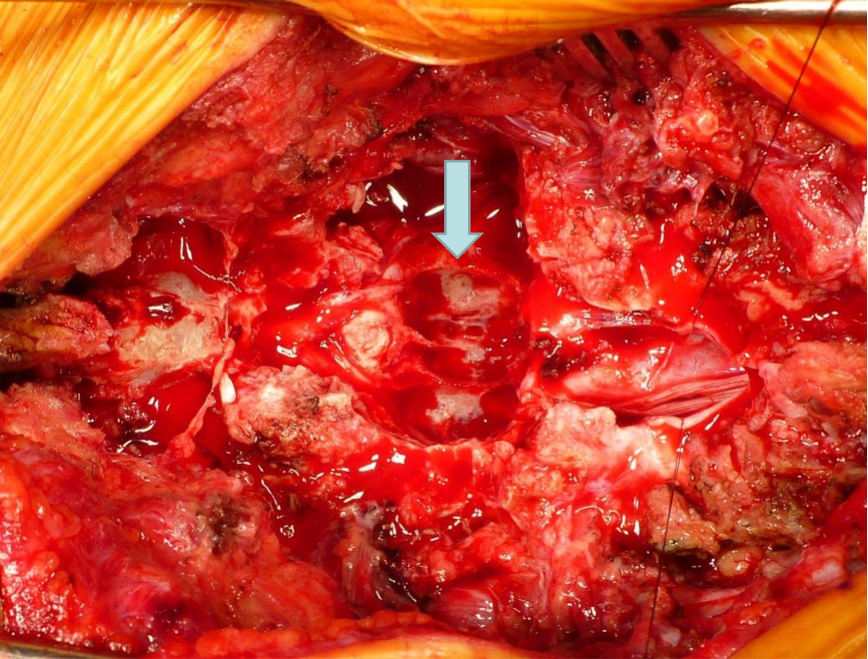
**Intra-operative photograph obtained after tumor resection.** During the patient’s surgery, we found extensive scalloping of the vertebral body and a large defect in the ventral dura mater due to tumor invasion. The arrow identifies the scalloping of the vertebral body.

**Figure 4:**
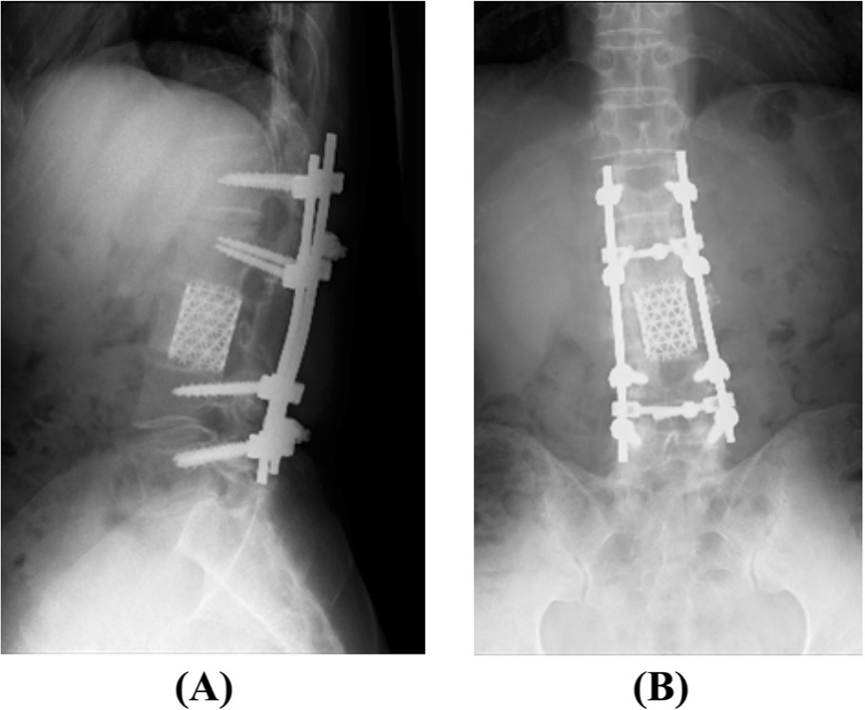
**Post-operative lateral (A) and anteroposterior (B) X-ray of the lumbar spine.** Circumferential fusion was performed using a titanium cage and pedicle screws.

A 42-year-old Japanese man presented to our department with the chief complaints of gait disturbance and muscle weakness in the left lower limb of a few months’ duration. He had no previous medical or surgical history. A physical examination performed at the first visit revealed that his muscle strength was grade 2-3/5 in the proximal portion of the left lower limb. He had full muscle strength in his right lower limb and the distal portion of his left lower limb. In addition, we observed a mild urinary disturbance. X-rays and computed tomography (CT) myelography images of the lumbar spine showed large scalloping of the L3 vertebral body (Figure 1). Magnetic resonance imaging (MRI) of the lumbar spine demonstrated a large soft-tissue mass in the spinal canal at L2-L3 and in the vertebral body at L3 (Figure 2). Surgery was performed to treat the spinal lesion. During the first step, laminectomy at L2-L3 with left L2-L3 total facetectomy was performed. In the next step, the dura mater was opened, and the tumor was completely resected at the branch of the two nerve roots entering the tumor with the assistance of combined motor-evoked potentials (MEPs) and somatosensory-evoked potentials (SEPs). After resecting the tumor, we found a large defect in the ventral dura mater due to tumor invasion and observed that most of the nerve roots ran laterally in the spinal canal due to tumor compression (Figure 3). In the third step, circumferential fusion was performed. Anterior vertebral body reconstruction was carried out using a titanium cage placed to treat the scalloping of the L3 vertebral body via the posterior transdural approach through the ventral defect in the dura mater. Right-sided posterolateral fusion was added at L1-L5 using instrumentation, including pedicle screws (Figure 4). In the final step, duraplasty was performed to reconstruct the resected tumor site using artificial dura mater. The pathological findings of the tumor were compatible with those of benign schwannoma. Although a mild sensory disturbance of the left lower limb persisted, the patient’s muscle strength in the proximal portion of the left lower limb recovered from 2-3/5 to 4-5/5, and we found that his gait status and urinary disturbance had resolved without local recurrence at the 2-year post-operative follow-up examination.

## Discussion

Spinal schwannoma is a representative benign intradural spinal tumor; however, giant lumbar schwannoma of the cauda equina is a rare pathology, and the treatment strategy, including the use of surgical procedures, for this condition is controversial. Kagaya *et al.* reported the case of a 57-year-old woman with a giant cauda equina schwannoma with scalloping of the posterior surface of the vertebral body from L3 to the sacrum in whom they performed laminectomy and incomplete resection of the tumor via the posterior approach [4]. Furthermore, they carried out posterior fusion from L2 to the sacrum, as well as L2-L3 posterolateral fusion with instrumentation and L3-S1 anterior fusion using bioactive ceramics and an autologous fibula graft. The patient remained almost symptom-free for 40 months after surgery. The authors concluded that the use of incomplete tumor resection because of the need to sacrifice many nerve roots can be considered a treatment option in cases of giant cauda equina tumor. However, incomplete tumor resection has also been reported to be associated with a risk of recurrence [12]. Therefore, we performed complete tumor resection for our patient with the assistance of combined MEPs and SEPs, taking into consideration the patient’s age.

Saito *et al.* reported the case of a 65-year-old woman with giant schwannoma of the cauda equina from T12 to L3 in whom they performed complete *en bloc* resection of the tumor without fusion, despite the fact that the tumor exhibited extensive scalloping of the vertebral bodies of L2 and L3 [6]. In that case, the patient’s pre-operative symptoms improved, and no vertebral fractures occurred during a post-operative course of 1.5 years. Furthermore, Le Corre *et al.* reported the case of a 49-year-old man with a giant spinal schwannoma at L3 and an extensive scalloping lesion of the L3 vertebral body. They performed complete resection of the tumor and one-stage circumferential fusion using pedicle screws and an expandable titanium cage to treat the vertebral body lesion via a posterior approach. The patient experienced no side effects of motor dysfunction, despite the presence of a sensory disturbance post-operatively [5].

We found that most of our patient’s nerve roots were compressed by the tumor and, as a result, had accumulated in the lateral spinal canal, with the exception of two nerve roots entering the tumor. Although we sacrificed the two nerve roots to perform complete tumor resection, the patient exhibited no severe neurological deficits after the surgery. Moreover, pre-operative CT myelography and MRI of the lumbar spine showed an extensive scalloping lesion at the L3 vertebral body. Therefore, we recognized the need to reconstruct the anterior vertebral body via a posterolateral approach, thus sacrificing an extensive lateral portion of L2 and L3, including L2-L3 facetectomy, or an anterior approach using a different skin incision pre-operatively. However, we confirmed the presence of accumulation of most of the cauda equina in the lateral spinal canal space in addition to a large defect in the ventral dura mater due to tumor invasion. Therefore, we placed a titanium cage in the L3 vertebral body lesion through the dural defect. This allowed us to perform anterior vertebral body reconstruction using a titanium cage via the posterior transdural approach. Hence, we think that the potential use of a posterior transdural approach for anterior vertebral body reconstruction should be taken into account in cases involving giant lumbar schwannomas of the cauda equina because the easy access to the vertebral body makes it possible to achieve circumferential fusion.

## Conclusions

We performed one-stage complete tumor resection and circumferential fusion using pedicle screws and a titanium cage via the posterior approach in a patient with giant cauda equina schwannoma with extensive scalloping of the vertebral body and achieved a good outcome post-operatively.

## Consent

Written informed consent was obtained from the patient for publication of this case report and any accompanying images. A copy of the written consent is available for review by the Editor-in-Chief of this journal.

## Abbreviations

CT: Computed tomography
MRI: Magnetic resonance imaging.
